# The neutrophil-to-lymphocyte ratio is associated with in-hospital heart failure in patients with ST-segment elevation myocardial infarction treated with primary percutaneous coronary intervention

**DOI:** 10.1016/j.heliyon.2024.e39761

**Published:** 2024-10-24

**Authors:** An-Cheng Hou, Jian-Ye Zhao, Yan-Jin Wei, Zhi-Hong Ou, Cun-Fei Liu

**Affiliations:** Department of Cardiology, Linyi People's Hospital, Shandong Second Medical University, Linyi City, Shandong Province, 276000, China

**Keywords:** Neutrophil-to-lymphocyte ratio, ST-Segment elevation myocardial infarction, Heart failure, Primary percutaneous coronary intervention

## Abstract

**Objective:**

To explore the association between the neutrophil-to-lymphocyte ratio (NLR) and in-hospital heart failure (HF) in patients with ST-segment elevation myocardial infarction (STEMI) undergoing primary percutaneous coronary intervention (PCI).

**Methods:**

Our present study included 413 patients diagnosed with STEMI and treated with primary PCI. We performed logistic regression models to evaluate the relationship between the NLR and in-hospital HF risk in subjects diagnosed with STEMI.

**Results:**

The incidence of HF after STEMI increased significantly with increasing NLR tertiles (the incidences of the first, second, and third tertiles were 5.07 %, 13.04 %, and 23.36 %, respectively; P < 0.001). Multivariate logistic regression model showed that elevated NLRs significantly increased the risk of in-hospital HF after adjusting for multiple potential covariates. The risk of HF in the second and third NLR tertile groups was 1.27 times greater (95 % CI, 0.42–3.92) and 3.09 times greater (95 % CI, 1.06–9.02) than that in the first tertile group (P for trend = 0.04). Moreover, the in-hospital HF risk increased by 58 % with per 1-SD increment in the NLR (OR, 1.58; 95 % CI 1.24–2.03; P < 0.001).

**Conclusions:**

Our study demonstrated that the NLR is positively correlated with in-hospital HF risk and is an independent predictor for in-hospital HF in STEMI subjects.

## 1 Introduction

Heart failure (HF) is a common complication of acute myocardial infarction (AMI), and patients with HF complicated with AMI have a poorer prognosis than those without HF [1,2]. To date, HF is still a serious complication of AMI, even with significant advances in coronary revascularization and drug treatment [[3], [4], [5]]. In-hospital HF can significantly increase the risk of early death in patients with acute myocardial infarction; the in-hospital mortality rate was 79 % greater in the HF group than in the non-HF group [2]. Therefore, timely identiﬁcation and intervention of AMI patients at high risk for HF development are expected to decrease major adverse cardiovascular events [6,7].

The neutrophil-to-lymphocyte ratio (NLR) is a novel biomarker of inflammation and has been found to be a predictor of major adverse cardiovascular events, such as cardiovascular mortality, all-cause mortality, in-stent thrombosis, and no reflow, in patients with STEMI undergoing primary PCI [[8], [9], [10], [11], [12], [13]]. However, its relationship with in-hospital HF complicating STEMI is still unclear, and only a few studies with small sample sizes have investigated this issue [14,15]. Moreover, no quantitative analysis of the NLR in patients with HF after STEMI was performed in these studies. Therefore, we conducted this study to evaluate the precise correlation between the NLR and in-hospital HF in patients with STEMI treated with primary PCI.

## 2 Methods

### 2.1 Study populations

In our present study, we retrospectively analysed patients who were first diagnosed with STEMI between January 2019 and December 2020 in the cardiac care unit of Linyi People's Hospital. STEMI was deﬁned based on the fourth universal deﬁnition of myocardial infarction [16]. All included patients were treated with primary PCI according to clinical practice guidelines [17], and only infarct-related arteries in STEMI patients were treated. Patients with a known history of old myocardial infarction, heart failure, active infections, malignancy or previous history of coronary intervention were excluded. After initial elimination of 108 patients according to the above criteria, 448 patients were enrolled in our study. Thirty-five patients were excluded because only coronary angiography was performed for different reasons, for example, coronary spasm or coronary dissection. Ultimately, 413 subjects with STEMI who underwent primary PCI were enrolled in our study. The study was approved by the Ethics Committee of Linyi People's Hospital (No YX200312), and written informed consent from patients was waived because the study was retrospective with anonymized data.

The primary endpoint was new-onset HF during hospitalization based on the Killip classification [18] (grade I: no signs of heart failure; grade II: lung rales <50 % of the lungs; grade III: acute pulmonary oedema; grade IV: presentation with cardiogenic shock). Patients with a Killip score ≥ II were defined as having HF.

### 2.2 Data and laboratory Measurements

The primary PCI operations were performed by experienced interventional cardiologists. Surgical information, including first medical contact (FMC) time, infarct-related artery, number of vessel lesions (coronary lumen stenosis >50 %), number of implanted stents, and postprocedural TIMI flow grade [19], was obtained from the medical records. In addition, demographic data, clinical information (history of hypertension, diabetes, dyslipidaemia, smoking and drinking, etc.) and laboratory data were also collected. Fasting venous blood samples were obtained at admission, and routine serum biochemical indicators were tested by an automatic biochemical analyser according to standard methods. Haematologic parameters, including neutrophil and lymphocyte counts, were tested, and then the NLR was calculated. All enrolled subjects were received transthoracic doppler echocardiography examinations in the first 24 h after admission.

### 2.3 Statistical analysis

Quantitative variables were first tested for a normal distribution with the Shapiro‒Wilk test and are summarized as the means ± SDs or medians with IQRs. Student's *t*-test or the Mann‒Whitney test was chosen for normally distributed or skewed data between two groups. One-way ANOVA or the Kruskal‒Wallis test was chosen for comparisons between multiple groups. Qualitative variables are presented as numbers and percentages. The chi-square test was chosen for comparisons between qualitative data. Tertiles of NLR were computed and then were analysed with logistic regression models. Univariable and multivariable logistic regression models were used to estimate the relationship between the NLR and in-hospital HF risk. The following covariables were adjusted in the most adjusted Multivariable analyses: gender, age, C-reactive protein, first medical contact time, hypertension history, diabetes history, alanine aminotransferase, creatinine, culprit vessel and number of lesions. In addition, the sensitivity and specificity of the NLR and its optimal cut-off value in predicting risk of in-hospital HF were performed with the receiver operating characteristic (ROC) curve analysis. A p value < 0.05 was considered to indicate statistical significance, and all the statistical analyses were carried out with SPSS software 18.0 (SPSS Inc., Chicago, IL, USA).

## 3 Results

### 3.1 Characteristics of enrolled patients

A total of 413 patients were ultimately enrolled in our current study. The main characteristics of the subjects are presented in Table 1. Fifty-seven patients developed HF during hospitalization, and the incidence of in-hospital HF was 13.8 % among all STEMI subjects. There was a significant difference in demographic characteristics between the HF group and the non-HF group. Generally, the people in the HF group were much older (mean age: 65.30 vs. 58.12 years, P < 0.001), and the proportion of women was much greater (45.61 % vs. 17.42 %, P < 0.001). Moreover, the incidence of diabetes (40.35 % vs. 19.10 %, P < 0.001), hypertension (66.67 % vs. 41.29 %, P < 0.001) and in-hospital mortality (10.53 % vs. 1.12 %, P < 0.001) in the HF group significantly increased.

**Table 1:** Characteristics of the study patients.

	HF(n = 57)	Non-HF(n = 356)	P value
Age(year)	65.30 ± 10.94	58.12 ± 12.80	<0.001
Female(%)	26(45.61 %)	62(17.42 %)	<0.001
Diabetes mellitus(%)	23(40.35 %)	68(19.10 %)	<0.001
Hypertension(%)	38(66.67 %)	147(41.29 %)	<0.001
SBP(mmHg)	121 ± 19.65	124.72 ± 18.27	0.159
DBP(mmHg)	76.23 ± 11.31	79.85 ± 13.31	0.052
Smoking(%)	21(36.84 %)	192(53.92 %)	0.041
Drinking(%)	6(10.53)	131(36.80)	<0.001
Death during Hospitalization(%)	6(10.53 %)	4(1.12 %)	<0.001
Hospitalization day	11.05 ± 6.13	8.32 ± 2.87	<0.001
LVEF(%)	47.00 ± 7.73	53.10 ± 5.71	<0.001
CRP(mg/l)	16.69(5.23–39.85)	4.31(3.10–9.69)	<0.001
NT-pro BNP(pg/ml)	2841(1269–5624)	780(260.1537)	<0.001
TnT(ng/ml)	4.13 ± 0.83	4.05 ± 0.76	0.482
WBC(10^9/L)	13.33 ± 4.87	10.08 ± 3.05	<0.001
ALT(IU/L)	55.95(34.25–86.5)	36.70(25.85–56.38)	<0.001
Neutrophil cells(10^9/L)	11.14 ± 4.55	7.88 ± 2.91	<0.001
Lymphocyte	1.48 ± 0.59	1.61 ± 0.97	0.353
NLR	8.74 ± 5.76	6.19 ± 4.40	<0.001
FBG(mmol/L)	10.23 ± 3.99	7.27 ± 2.89	<0.001
Cr(mmol/L)	76.36 ± 28.15	66.64 ± 17.04	0.014
TG(mmol/L)	1.39 ± 0.65	1.72 ± 1.49	0.116
TC(mmol/L)	4.57 ± 1.18	4.69 ± 1.18	0.504
LDL-c(mmol/L)	3.08 ± 1.10	3.07 ± 0.89	0.925
HDL-c(mmol/L)	1.12 ± 0.26	1.05 ± 0.26	0.211

Abbreviation: SBP: systolic blood pressure; DBP: diastolic blood pressure; LVEF: left ventricular ejection fraction; CRP: C-reactive protein; WBC: white blood cell; ALT: alanine aminotransferase; FBG: fasting blood glucose; TG: triglyceride; TC: total cholesterol; LDL-c: low-density lipoprotein cholesterol; HDL-c: high-density lipoprotein cholesterol.

The levels of the main laboratory indicators are also presented in Table 1. The levels of CRP, NT-pro BNP, and leukocyte and neutrophil counts were greater in the HF group than that in the non-HF group. The mean NLR of individual subjects was significantly greater in the HF group than in the non-HF group (8.74 vs. 6.19, P < 0.001). The lipid levels and peak troponin I levels were comparable between the two groups. Our echocardiography data showed that the left ventricular ejection fraction (LVEF) of the HF group was much lower than the non-HF group (47.00 % vs. 53.10 %, P < 0.001).

### 3.2 Correlation analysis of the NLR with clinical parameters

The associations between the NLR and clinical parameters were explored with Pearson's or Spearman's correlation coefficients (Table 2). The NLR was positively associated with creatinine (r = 0.133, P = 0.007), ALT (r = 0.262, P < 0.001), CRP (r = 0.141, P = 0.006) and NT-pro BNP (r = 0.151, P = 0.003). However, the NLR was negatively related to the LVEF (r = −0.204, P < 0.001) and number of lesions (r = −0.172, P < 0.001).

**Table 2:** Correlation coeﬃcients between NLR with clinical parameters.

	’Coefficients	P value
Age	0.028	0.568
Creatinine	0.133	0.007
ALT	0.262	<0.001
CRP	0.141	0.006
NT-pro BNP	0.151	0.003
LVEF	−0.204	<0.001
Number of lesions	−0.172	<0.001

Abbreviation: ALT: alanine aminotransferase; CRP: C-reactive protein; LVEF: left ventricular ejection fraction.

### 3.3 Coronary angiography and procedure characteristics

The main characteristics of the surgery-related data were shown in Table 3. The time of first medical contact in the HF group was much longer than that in the non-HF group. Moreover, the HF group was more likely to have left coronary artery-related STEMI and multivessel disease. However, there was no significant difference in the number of implanted stents between the HF group and the non-HF group.

**Table 3:** Characteristics of procedural information.

	HF(n = 57)	Non-HF(n = 356)	P value
First medical contact (h)	5.70 ± 4.05	4.59 ± 2.88	0.012
Number of lesions			0.008
1	22(38.60 %)	213(59.83 %)	
2	29(50.88 %)	125(35.11 %)	
3	6(10.52 %)	18(5.06 %)	
Number of stents	1.7 ± 0.4	1.4 ± 0.3	0.203
Postprocedural TIMI flow grade<3(%)	2(3.51 %)	14(3.93 %)	0.881
Culprit vessel			<0.001
LM(%)	5(8.77 %)	2(0.56 %)	
LAD(%)	39(68.42 %)	176(50 %)	
LCX(%)	2(3.51 %)	37(10.39)	
RCA(%)	11(19.30 %)	141(39.61 %)	

Abbreviation: TIMI: Thrombolysis in Myocardial Infarction; LM: left main artery; LAD: left anterior descending artery; LCX: left circumflex artery; RCA; right coronary artery.

### 3.4 Baseline clinical characteristics of subjects according to the NLR

We further compared the clinical characteristics of STEMI patients based on NLR tertiles (Table 4). The incidence of HF was 5.07 % in the first NLR tertile and increased significantly to 23.36 % in the third NLR tertile. Significant differences were also found in the levels of NT-pro BNP, CRP, white blood cells, etc., between different NLR tertiles. A more detailed comparison is shown in Table 4.

**Table 4:** Characteristics of the study patients according to NLR tertiles.

	Q1(n = 138)	Q2(n = 138)	Q3(n = 137)	P value
NLR	≤4.17	4.17-6.69	>6.69	
Age(year)	56.90 ± 12.84	60.14 ± 13.47	60.30 ± 11.81	0.044
Female(%)	27(19.57 %)	27(19.57 %)	34(24.82 %)	0.471
Heart Failure(%)	7(5.07 %)	18(13.04 %)	32(23.36 %)	<0.001
Diabetes mellitus(%)	27(19.57 %)	32(23.19 %)	32(23.36 %)	0.680
Hypertension(%)	47(34.06 %)	77(55.80 %)	61(44.53 %)	0.001
SBP(mmHg)	126.26 ± 16.29	122.57 ± 20.38	123.78 ± 18.52	0.240
DBP(mmHg)	79.80 ± 12.17	78.42 ± 13.22	79.83 ± 13.91	0.594
Smoking(%)	78(56.52 %)	75(54.35 %)	60(43.80 %)	0.228
Drinking(%)	50(36.23 %)	50(36.23 %)	37(27.01 %)	0.188
Death during Hospitalization(%)	2(1.45 %)	2(1.45 %)	6(4.38 %)	0.189
Hospitalization day(day)	7.93 ± 2.90	8.71 ± 3.50	9.44 ± 4.23	0.003
LVEF(%)	53.85 ± 5.76	52.29 ± 5.73	50.75 ± 7.13	<0.001
CRP(mg/l)	3.67(3.10–11.37)	4.61(3.10–9.83)	6.10(3.10–17.06)	0.015
NT-pro BNP(pg/ml)	699.85(380.35–1450.50)	1043.00(373.85–1884.00)	1251.00(602.20–2501.50)	0.001
TnT(ng/ml)	4.08 ± 0.72	4.16 ± 0.88	4.09 ± 0.82	0.658
WBC(10^9/L)	8.94 ± 2.47	10.28 ± 2.79	12.38 ± 4.22	<0.001
ALT(IU/L)	31.55(23.53–50.05)	38.50(26.98–59.13)	48.50(32.80–75.50)	<0.001
FBG(mmol/L)	6.96 ± 2.91	7.32 ± 2.96	8.77 ± 3.49	<0.001
Cr(mmol/L)	65.74 ± 15.89	67.40 ± 19.29	70.82 ± 21.81	0.082
TG(mmol/L)	1.99 ± 1.72	1.79 ± 1.53	1.25 ± 0.64	<0.001
TC(mmol/L)	4.74 ± 1.18	4.71 ± 1.32	4.56 ± 1.02	0.380
LDL-c(mmol/L)	3.09 ± 1.02	3.08 ± 0.91	3.03 ± 0.84	0.878
HDL-c(mmol/L)	1.02 ± 0.25	1.05 ± 0.26	1.10 ± 0.28	0.046

Abbreviation: SBP: systolic blood pressure; DBP: diastolic blood pressure; LVEF: left ventricular ejection fraction; CRP: C-reactive protein; WBC: white blood cell; ALT: alanine aminotransferase; FBG: fasting blood glucose; TG: triglyceride; TC: total cholesterol; LDL-c: low-density lipoprotein cholesterol; HDL-c: high-density lipoprotein cholesterol.

### 3.5 Association between the NLR and in-hospital HF risk Constructed by STEMI

We performed univariable and multivariable logistic regression analyses to evaluate the correlation between the NLR and HF risk in STEMI patients. The results are presented in Table 5. The unadjusted ORs of HF risk were 2.81 times greater (OR: 2.81; 95 % CI: 1.13–6.96) and 5.70 times greater (OR: 5.70; 95 % CI: 2.42–13.44) than those in the bottom NLR tertile group (p for trend <0.001). Multivariate logistic regression models showed that the ORs of HF risk were significantly increased in the second and third NLR tertiles. For example, the age- and sex-adjusted ORs were 2.63 (OR: 2.63; 95 % CI: 1.04–6.68) and 5.41 (OR: 5.41; 95 % CI: 2.25–13.03) in the second and third NLR tertile groups, respectively (p for trend <0.001). According to the most adjusted model (adjusted for sex, age, first medical contact time, hypertension history, diabetes history, alanine aminotransferase, creatinine, culprit vessel and number of vessels), the adjusted ORs were 1.27 (OR: 1.27; 95 % CI: 0.42–3.92) and 3.09 (OR: 3.09; 95 % CI: 11.06-9.02) in the second and third NLR tertile groups, respectively (P for trend = 0.04). In addition, each 1 standard deviation increment in the NLR was associated with a 1.58-fold increase in the risk of in-hospital HF (OR: 1.58; 95 % CI: 1.24–2.03; P < 0.001).

**Table 5:** Odds ratios (95 % CIs) of HF according to tertiles of NLR.

	Q1	Q2	Q 3	P for trend
NLR	≤4.17	4.17-6.69	>6.69	
Case/number at risk	7/138	18/138	32/137	<0.001
Odd Ratios (95%CI)				
Model 1	1	2.81(1.13–6.96)	5.70(2.42–13.44)	<0.001
Model 2	1	2.63(1.04–6.68)	5.41(2.25–13.03)	<0.001
Model 3	1	2.03(0.77–5.32)	3.34(1.31–8.55)	0.01
Model 4	1	1.27(0.42–3.92)	3.09(1.06–9.02))	0.04
Per 1SD NLR increment		1.58(1.24–2.03)		<0.001

Model 1: unadjusted OR.

Model 2: adjusted for gender and age.

Model 3: adjusted for gender, age, hypertension history, diabetes history, alanine aminotransferase and creatinine.

Model 4: adjusted for gender, age, C-reactive protein, first medical contact time, hypertension history, diabetes history, alanine aminotransferase, creatinine, culprit vessel and number of lesions.

ROC curve analysis revealed that the area under the curve was 0.676 (95 % CI: 0.629–0.721; P < 0.001) for the exact association between the NLR and HF. The optimal cut-off value of the NLR for predicting in-hospital HF incidence was 6.58, with a sensitivity of 59.65 % and a specificity of 69.10 %.

## 4 Discussions

Although clinical outcomes have significantly improved with the development of drug therapy and coronary revascularization in patients with HF after STEMI, the incidence of HF complicating STEMI is still relatively high and is associated with increased mortality and a poor prognosis [1,2]. Recent epidemiological studies and meta-analyses have demonstrated that the NLR is positively associated with in-hospital and long-term adverse events in patients with acute myocardial infarction [9,20]. However, few studies have focused on its correlation with in-hospital HF in STEMI subjects, especially in the era of coronary intervention. In our present study, we investigated the association between the NLR and in-hospital HF in STEMI patients undergoing primary PCI. The major findings of our present study are as follows. First, the incidence of HF increased from 5.07 % to 23.36 % with increasing NLR tertiles. Second, patients with HF complicated with STEMI had a substantially increased risk of mortality (10.53 % vs. 1.12 %, P < 0.001). Third, the NLR was positively correlated with the development of HF after STEMI and considered an independent predictor of in-hospital HF risk in STEMI patients.

Many clinical studies have demonstrated the role of inflammatory mediators in the diagnosis and prognosis of HF over the past decade. For example, high-sensitivity CRP, soluble growth stimulation expressed gene 2 (sST2) and galectin-3 are clinically useful in risk stratification for chronic and acute HF patients [21]. These inflammatory biomarkers were found to be associated with the mortality of HF patients. However, the exact mechanisms of inflammation in HF development and progression are not well elucidated. The NLR is an inexpensive and easily obtained haematological parameter. As a clinical marker of universal inflammation. Studies have demonstrated that the NLR is a superior indicator to other leukocyte subtypes or total white blood cell count in predicting cardiovascular disease [22]. Our present study also showed that an elevated NLR is related to a greater incidence of in-hospital HF in STEMI patients. The pathophysiological mechanism is still unclear. Several possible mechanisms are as follows. The inflammatory response plays an important role in the progression of AMI. An increase in the number of neutrophils in ischaemic areas after acute myocardial infarction represents an inflammatory reaction whereby neutrophils further facilitate monocyte adhesion and aggregation into ruptured plaques, thereby contributing to endothelial cell damage, oxidative stress and microvascular obstruction by releasing proteolytic enzymes or reactive oxygen species [[23], [24], [25]]. In addition, it has been found that neutrophils contribute to myocardial fibrotic scar formation, which has an adverse impact on left ventricular systolic function [26]. Moreover, a low lymphocyte count in cardiovascular disease patients represents physiological stress and is associated with poor outcomes [27]. The NLR, which is accompanied by changes in neutrophils and lymphocytes during inflammation, is related to myocardial remodelling after ischaemia and reperfusion injury [28,29]. Accordingly, studies have confirmed that a high NLR is associated with decreased left ventricular ejection fraction in STEMI patients [28,[30], [31], [32]]. Moreover, a prospective study has demonstrated that a higher NLR was related to increased mortality in acute decompensated HF patients [33]. Similarly, we also observed a greater neutrophil count and lower lymphocyte count in the HF group than in the non-HF group in our study. The incidence of HF after STEMI significantly increased with increasing NLR tertiles (the incidence of HF in each NLR tertile was 5.07 %, 13.04 % and 23.36 %, respectively). Moreover, we also found that the NLR was negatively related to the LVEF (the correlation coefficient was −0.204, P < 0.001).

Our current study had several limitations. First, our study was an observational study, and the conclusions should be interpreted with caution. We included only STEMI patients, and we did not analyse other acute coronary syndrome patients, such as non-ST-elevation myocardial infarction (NSTEMI) patients and unstable angina patients. Our present study cannot determine causality between the NLR and in-hospital HF because of the cross-sectional study design. Second, although several potential covariates were adjusted for in our multiple logistic regression model, some residual covariates may still exist. Third, we only measured the NLR at admission and did not observe dynamic changes in the NLR with HF development. Finally, the long-term impact of the NLR on HF after discharge in STEMI patients was not evaluated.

## 5 Conclusions

In summary, our current study demonstrated that the NLR is positively related with in-hospital HF in STEMI patients treated with primary PCI and is an independent risk factor in predicting in-hospital HF in STEMI patients. As the NLR is an easily obtained and tested biochemical parameter, the NLR may have additional predictive value for in-hospital HF in STEMI subjects.

## CRediT authorship contribution statement

**An-Cheng Hou:** Writing – original draft, Methodology, Formal analysis, Data curation. **Jian-Ye Zhao:** Writing – original draft, Methodology, Formal analysis, Data curation. **Yan-Jin Wei:** Supervision, Methodology, Formal analysis. **Zhi-Hong Ou:** Writing – review & editing, Supervision, Formal analysis. **Cun-Fei Liu:** Writing – review & editing, Methodology, Formal analysis, Conceptualization.

## Data availability

Data will be made available on request

## Funding

The study was supported by a grant from the 10.13039/501100007129Natural Science Foundation of Shandong province (No ZR2020MH018).

## Declaration of Competing Interest

The authors declare the following financial interests/personal relationships which may be considered as potential competing interests: Cun-Fei Liu reports was provided by The Natural Science Foundation of Shandong province. If there are other authors, they declare that they have no known competing financial interests or personal relationships that could have appeared to influence the work reported in this paper.
